# Diffuse Alveolar Hemorrhage: An Unexpected Effect After Taking Acetylsalicylic Acid

**DOI:** 10.7759/cureus.21486

**Published:** 2022-01-22

**Authors:** Mariana M Gomes, Carolina Barros, Helena Luís, Mariana Bilreiro, Bela Machado

**Affiliations:** 1 Internal Medicine, Hospital Central do Funchal - Serviço de Saúde da Região Autónoma da Madeira, Entidade Pública Empresarial da Região Autónoma da Madeira (SESARAM, EPERAM), Funchal, PRT

**Keywords:** hemoptysis, dyspnea, acetylsalicylic acid, antiplatelet therapy, diffuse alveolar hemorrhage

## Abstract

Diffuse alveolar hemorrhage (DAH) is a rare, acute, and life-threatening condition that in most cases is associated with pulmonary-renal syndromes, connective tissue disorders, infections, and drugs. We report a case of a 45-year-old male who developed a diffuse pulmonary hemorrhage after taking 500 mg of acetylsalicylic acid for a month in the context of acute lower back pain. The prolonged use of this acetylsalicylic acid dose led to an increased risk of bleeding. This report describes a rare bleeding site that clinicians should be aware of when managing patients who were exposed to prolonged high dose acetylsalicylic acid.

## Introduction

Diffuse alveolar hemorrhage (DAH) is a life-threatening condition that clinically presents with hypoxemic respiratory failure, hemoptysis, and a drop in hematocrit and diffuse pulmonary infiltrates [1,2]. Most cases of DAH are caused by a small number of conditions such as pulmonary-renal syndromes, connective tissue disorders, infections, and drugs [1].

The treatment of DAH depends on the underlying cause, and in most cases, corticosteroid therapy is the treatment of choice. In cases of drug-induced DAH or caused by other exposures, it is recommended to discontinue the drug [1].

## Case presentation

A 45-year-old male patient, professional driver, smoker (38-pack year history of cigarette use), with no other known medical history, was admitted to the emergency department with one-day history of worsening dyspnea, hemoptysis, and pleuritic chest pain. The patient denied other symptoms.

On examination, the patient was pale and dyspneic with difficulty completing sentences. Blood pressure was normal (120/63 mmHg) with a heart rate of 127 beats per minute, and the temperature was 36.1ºC. Pulmonary auscultation revealed decreased vesicular murmur and bilateral crackles. The arterial blood analysis showed a pH of 7.48, partial pressure of carbon dioxide (PaCO2) of 23.2 mmHg (3.09 kPa), partial pressure of oxygen (PaO2) of 42.9 mmHg (5.72 kPa), oxygen saturation of 82%, bicarbonate (HCO3) 19.9 mmol/L, Hb 4.9 g/dL, and lactate 4.5 mmol/L. 

**Figure 1:**
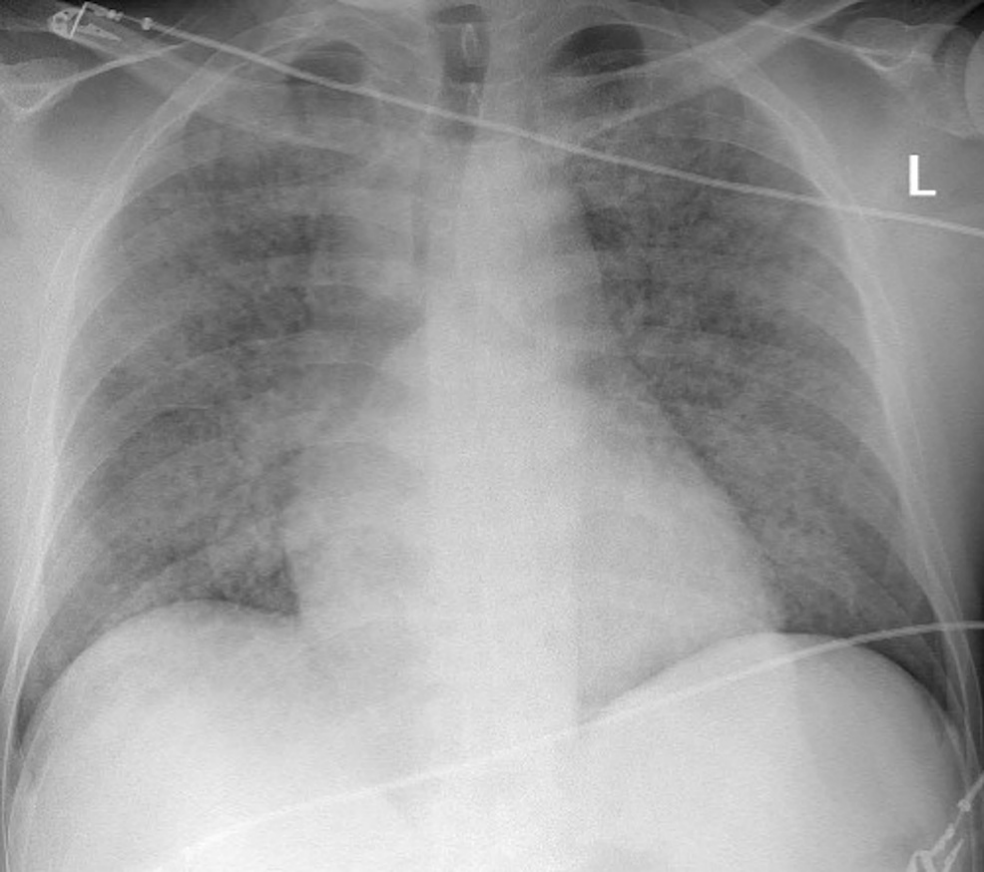
Anteroposterior chest X-ray performed at admission showing bilateral infiltrations suggesting diffuse alveolar hemorrhage

The electrocardiogram showed sinus tachycardia and the chest X-ray revealed a diffuse reticulonodular pattern (Figure 1).

**Figure 2:**
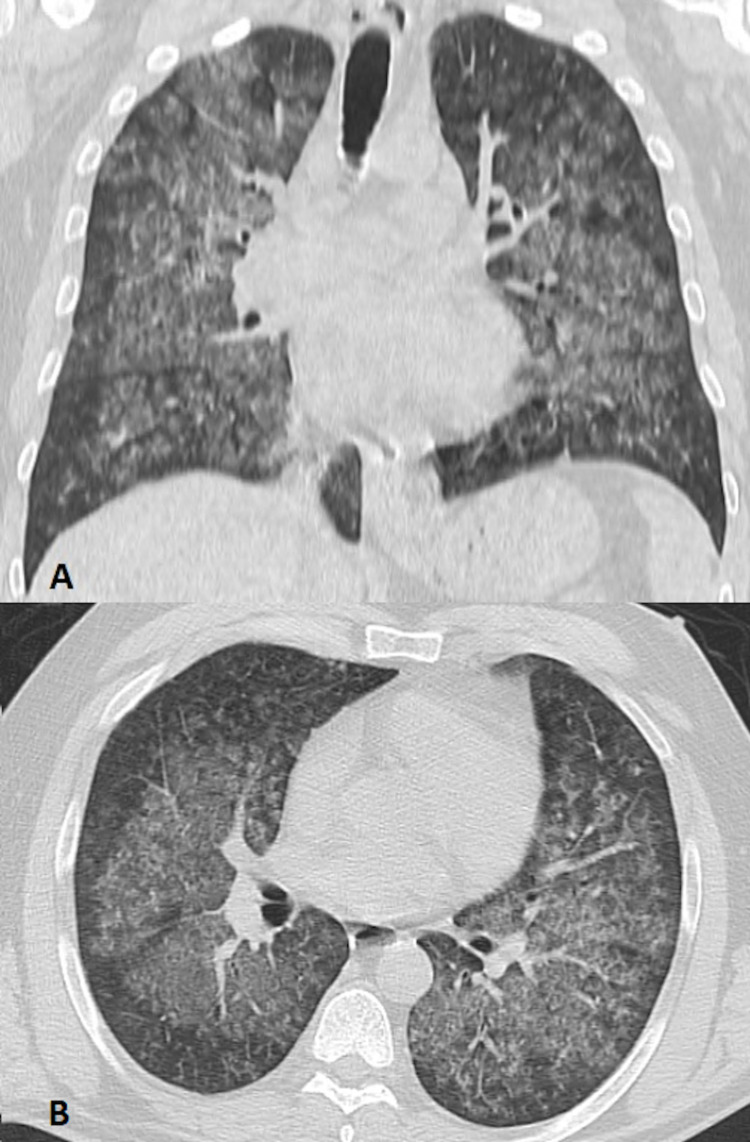
Coronal (A) and transverse (B) sections of CT of the chest with bilateral dense infiltrates on the day of admission

A computed tomography of the chest showed diffuse cotton-wool infiltration of the lung parenchyma, sparing the peripheral parenchyma, suggesting hemorrhagic infiltration. There were no signs of pulmonary embolism (Figure 2).

Laboratory findings are described in Table 1. The patient had acute and severe anemia with a hemoglobin of 5.2 g/dL (previous value of 17.1 g/dL), hematocrit of 18.9% (previous value of 49.7%), leukocytosis, with a normal platelet count. The coagulation profile reported a prolonged prothrombin time (PT) with a normal international normalized ratio (INR) and normal D-dimers levels. Creatinine levels were slightly above the upper limit and C-reactive protein was increased. Serum protein electrophoresis and urinalysis was normal. Urine and serum toxicology were negative.

**Table 1:** Laboratory analyses INR: International normalized ratio; PT: Prothrombin time; LDH: Lactate dehydrogenase; TIBC: Total iron-binding capacity

Parameter	On admission	2^nd^ day of hospitalization	9^th^ day of hospitalization	Reference values
Hemoglobin (g/dL)	5.2	6.3	8.8	13.7-17.3
Hematocrit (%)	18.9	21	29.4	40-51
Leukocytes (10³xµL)	18.3	16.5	9.7	4.2-10.8
Platelets (10³xµL)	382	273	347	144-440
INR	1.1	-	0.9	0.9-1.2
PT (s)	14.1	-	11.6	9.4-12.5
D-dimers (ng/mL)	175	-	-	<255
Creatinine (mg/dL)	1.25	0.9	0.94	0.7-1.2
LDH (U/L)	297	384	196	0-246
Total bilirrubin (mg/dL)	-	1.55	1.1	0.3-1.2
Indirect bilirrubin (mg/dL)	-	1.27	-	0-1.1
C-reactive protein (mg/L)	15	59	1.8	<6.1
Sedimentation rate (mm)	13	-	9	0-15
Iron (µg/dL)	-	-	12	45-182
Transferrin (mg/dL)	-	-	333	200-360
TIBC (mg/dL)	-	-	416	250-450
Transferrin saturation (%)	-	-	2.9	20-50
Ferritin (ng/mL)	-	-	93.6	30-400

Given the clinical suspicion of DAH, supportive treatment was given and methylprednisolone 1 g daily was started. He did supplemental oxygen with 4 liters/min for one day with progressive weaning and was treated with four units of red blood cell transfusion. Further investigations were performed, such as serologic tests (HIV, hepatitis B, and C), autoimmune workup including antinuclear antibody, anti-double‐stranded DNA (anti‐dsDNA), anti-glomerular basement membrane antibodies, antineutrophilic cytoplasmic autoantibodies, and rheumatoid factor, which all were negative. After these findings, corticosteroid therapy was discontinued.

When asked about drug history, the patient mentioned taking 500 mg of acetylsalicylic acid (Aspirin®) for one month in the context of acute low back pain. In the absence of other findings; the patient was diagnosed with DAH secondary to acetylsalicylic acid.

During hospitalization, chest imaging and hemoglobin levels slowly improved, and the patient no longer needed oxygen supplementation.

One month after hospital discharge, the patient had no complaints, normal chest radiograph, and pulmonary auscultation, with hemoglobin back to the normal level.

## Discussion

DAH is a diagnosis to consider when a patient presents with hypoxemia, new-onset anemia, and an alveolar infiltrate on a chest X-ray. About one-third of patients will not experience hemoptysis [1]. A detailed medical history, including drug exposure, physical examination, and targeted laboratory evaluation often suggests the underlying cause [2].

An abnormal urinalysis or elevated blood urea nitrogen and serum creatinine can occur as a manifestation of pulmonary-renal syndromes such as granulomatosis with polyangiitis and Goodpasture syndrome [3]. In these cases, a kidney biopsy should be performed to identify the underlying cause and start appropriate therapy [2].

When the diagnosis is not apparent, to confirm the diagnosis, an early bronchoscopy with bronchoalveolar lavage is required and blood should be present on three sequential lavage aliquots from the affected area of the lung. These specimens should be sent for routine bacterial, mycobacterial, fungal, and viral studies to rule out infection [3]. 

DAH is a medical emergency and in most cases is treated with corticosteroid and immunosuppressive therapy [3]. In cases of drug-induced DAH, it is recommended to discontinue the drug [1]. Many drugs have been associated with DAH, including anticoagulants [4-6], thrombolytic agents [7], and antiplatelet agents [8-10]. Aspirin® has traditionally been used as an analgesic and anti-inflammatory drug. A low dose is used in secondary and primary prevention of cardiovascular events, associated with an increased risk of gastrointestinal and intracranial hemorrhage [11,12], however, there is no data reporting cases of DAH associated with its use alone. In this case, supportive care was given, the acetylsalicylic acid was discontinued, and the patient achieved a good clinical response.

## Conclusions

Drug-induced DAH has been associated with fibrinolytic therapy, oral anticoagulation, and dual antiplatelet therapy. In this case, no other etiological factor for DAH was identified, apart from the prolonged use of a high-dose acetylsalicylic acid. As acetylsalicylic acid is a medication that is commonly used and easily accessible, clinicians should be more aware of its risks and complications when taken inappropriately which can cause life-threatening conditions.
